# Predicting the Potential Distribution of *Lagotis* Medicinal Plants on the Qinghai‐Xizang Plateau, With a Maximum Entropy Model

**DOI:** 10.1002/ece3.71319

**Published:** 2025-04-23

**Authors:** Huiyuan Ma, Bo Wang, Xue Yang, Guoying Zhou

**Affiliations:** ^1^ College of Life Sciences Qinghai Normal University Xining China; ^2^ CAS Key Laboratory of Tibetan Medicine Research Northwest Institute of Plateau Biology Xining China

**Keywords:** climate change, dominant factors, *Lagotis*, maximum entropy model (MaxEnt), potential distribution, Qinghai‐Xizang plateau

## Abstract

*Lagotis* is a Tibetan medicine with high medicinal and research value in the Qinghai‐Xizang Plateau region. Predicting its potential suitable areas can provide important references for the protection and research of *Lagotis*. This study investigated and collected 769 site data of six species of *Lagotis* and 28 environmental factor data, and used the maximum entropy model (MaxEnt) to simulate the distribution of suitable areas under three climate scenarios (RCP2.6, RCP6.0, RCP8.5) in the 2050s and 2070s. The results showed that: (1) The AUC values of the model training dataset for the six *Lagotis* species were all > 0.94, indicating that the predictions of the model were accurate. (2) Altitude (alt), isothermality (bio3), and average annual precipitation (bio12) were the main environmental factors affecting the geographical distribution of *Lagotis*. (3) Under the current climate conditions, with the increase of greenhouse gas emission concentration, the suitable distribution areas for the *Lagotis* genus plants are primarily located in the eastern, central, and southern parts of the Qinghai‐Xizang Plateau. (4) Compared to the present period, the suitable areas of *Lagotis integra*, *Lagotis angustibracteata*, and *Lagotis ramalana* potential distribution area will expand in the future. The area of high suitability for *Lagotis macrosiphon* shows an increasing trend. The area of moderate suitability for *Lagotis brachystachya* is decreasing, while the area of high suitability is increasing. In contrast, the suitable area of *Lagotis brevituba* decreased greatly. (5) The study found that *Lagotis* genus plants are suitable for survival in high‐altitude, low‐temperature areas. The research findings provide a basis for the cultivation and breeding of medicinal plants in the Qinghai‐Xizang Plateau, the migration protection, and the construction of nature reserve communities.

## Graphical abstract

Lagotis is a Tibetan medicine with high medicinal and research value in the Qinghai‐Xizang Plateau region. Predicting its potential suitable areas can provide important references for the protection and research of Lagotis. This study investigated and collected 769 site data of 6 species of Lagotis and 28 environmental factor data and used the maximum entropy model (MaxEnt) to simulate the distribution of suitable areas under three climate scenarios (RCP2.6, RCP6.0, RCP8.5) in the 2050s and 2070s.

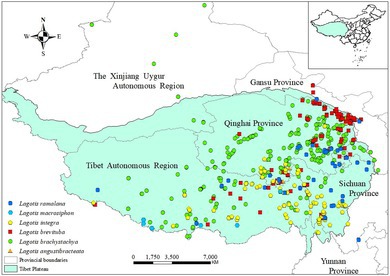

## 1 Introduction

In the 21st century, the sustainability of the global ecosystem has been seriously threatened by the combined effects of global warming, socio‐economic development, and ecological changes (Matesanz and Valladares 2014; Thuiller 2014). Climate change is one of the primary drivers affecting the distribution of species and the layout of biodiversity, with particularly significant impacts on the plant kingdom (Zhao et al. 2013). The synergistic changes in temperature and precipitation have a crucial impact on the reproduction and survival of vegetation, especially under extreme environmental conditions (Nippert et al. 2009). The Qinghai‐Xizang Plateau, known as the third pole of the Earth, has an average altitude of over 4000 m. Since 1960, the overall climate warming and humidification of the Qinghai‐Xizang Plateau has occurred, with an average annual temperature increase of 0.35°C per decade, more than twice the global warming rate during the same period (Tao et al. 2014). Along with the rising temperatures, precipitation has also shown an increasing trend. Research indicates that the annual precipitation on the Qinghai‐Xizang Plateau has increased by an average of 7.9 mm per decade. The cold and high‐altitude climate characteristics make the plants here more susceptible to the effects of climate change compared to other regions at the same latitude (Wang et al. 2020). Many unique species of the Qinghai‐Xizang Plateau are facing the serious threat of restricted growth and distribution areas, and even extinction, due to the deteriorating living environment (Hu et al. 2020). *Lagotis* is a perennial herb of the family Scrophulariaceae, commonly found on shady slopes of gravel belts, alpine meadows, and shrub grasslands at altitudes of 3500–4500 m (Zhu et al. 2019; Gong et al. 2022; Sun et al. 2011). As a type of Tibetan medicine, this plant is widely used in the Tibetan medical system and is a commonly used high‐quality medicinal material in Tibetan medicine (Fan et al. 2021). In recent years, due to the increase in market demand, the wild plant resources of *Lagotis* have gradually become scarce, leading to a sharp decline in its resource volume. Coupled with the increasing interference of climate change, its survival is under severe threat (Zhu et al. 2017). Under the background of climate change and human factors, how to protect and sustainably utilize the medicinal plant resources of *Lagotis* in the Qinghai‐Xizang Plateau region has become an urgent scientific issue that needs to be addressed. However, at present, the supply of *Lagotis* still comes from natural wild resources, and since most of the *Lagotis* grows in the gravelly areas of the shady slopes of the high mountains, its distribution area is narrow and dispersed, and it has great vulnerability. The harsh environmental conditions of high‐altitude mountains, combined with the narrow ecological niche adaptability of *Lagotis* species, render their cultivation an impractical supplementation approach. Therefore, combining manual surveys and models can be studied more easily (Lakey 2016). *Lagotis* is a species sensitive to environmental changes, and its distribution range and ecological niche are closely related to climatic conditions, so it can effectively reflect the impact of climate change on its living environment. In addition, the distribution data of *Lagotis* is relatively abundant, which provides a solid data base for simulation using the MaxEnt model. So we used the MaxEnt model to simulate and analyze its geographical distribution pattern and potential distribution area, to grasp and find out the dominant factors affecting its distribution, in order to protect the wild resources of *Lagotis* and to achieve the sustainable use of the resources. However, there are relatively few research reports on the suitable habitat distribution and future distribution of *Lagotis* in the Qinghai‐Xizang Plateau region.

Due to the ecological diversity and geographic complexity of the Qinghai‐Xizang Plateau, the species distributions of many plants are difficult to study, and medicinal plants have received more attention because of their often high economic value and ecological significance (Li, Su, et al. 2024; Li, Zhaxi, et al. 2024; Li, Ma, et al. 2024). Studies have shown that the distribution pattern of medicinal plants is not only constrained by the natural environment but also closely related to the intensity and extent of human activities (Alami et al. 2024). On the Qinghai‐Xizang Plateau, areas of high medicinal plant diversity often overlap with areas of high human activity, which further increases the complexity of research (Liu et al. 2021). Therefore, species distribution modeling is usually used for simulation and prediction. It is a very applicable and important tool for studying the spatial distribution of species under environmental and climate change (Liu et al. 2009; Wang, Yang, et al. 2019; Wang, Wu, et al. 2019; Kaky and Gilbert 2016). The maximum entropy model (MaxEnt) is one of the widely used predictive models in recent years (Phillips and Dudík 2008). The MaxEnt model is based on the theory of maximum entropy and simulates the possible distribution areas of target species through their actual distribution sites and a variety of environmental variables, especially in the case of a small number of species distribution sites, and unclear correlation of various environmental elements, but still can achieve accurate simulation and prediction results. This is a reflection of its robustness advantage (Renner and Warton 2013). At the technical level, by combining environmental variables and species distribution data, the MaxEnt model is able to effectively identify key climatic factors affecting species distribution and generate high‐resolution distribution probability maps (Ahmadi et al. 2023; Merow et al. 2013). It is computationally efficient and has relatively low data requirements and is able to make full use of the limited distribution point data for modeling (Radosavljević and Anderson 2014; Yi et al. 2016). MaxEnt models stand out for their excellent robustness and powerful technical features, especially when dealing with complex data and uncertainty (Kumar 2012). The core strength of the MaxEnt model is its adaptability and stability to the data, avoiding overfitting the data and being able to maintain reliable predictive performance (Lei and Xu 2010). It is commonly used in studies on the establishment of nature reserves, the prediction of potential habitats for invasive species, and the effects of climate change on species (Yang et al. 2013). Suo et al. (2024) utilized the MaxEnt model in conjunction with ArcGIS software to investigate the ecological suitability of *Forsythia suspensa* Thunb. Vahl within Shanxi Province; Yao et al. (2023) used the MaxEnt model to predict the potential distribution areas of noxious and miscellaneous weeds in Xinjiang under different climatic scenarios. In addition to this, Li, Su, et al. (2024), Li, Zhaxi, et al. (2024), Li, Ma, et al. (2024), She et al. (2021) and Dong et al. (2022) have also utilized the MaxEnt model to predict the dynamic changes in the future geographical distribution patterns of *Asterothamnus centraliasiaticus*, *Notopterygium incisum*, and *Stellera chamaejasme* on the Qinghai‐Xizang Plateau, as well as the significant environmental factors affecting their geographical distribution.

There are about 30 species in the *Lagotis* genus, which are mainly distributed in the northern hemisphere, including Central and North Asia and North America. In China, there are 17 species of the *Lagotis* genus, mainly from south‐west to north‐west (Li et al. 2014). The six species selected in this paper are representative of the Qinghai‐Xizang Plateau and are also the six basal source medicinal plants of the Tibetan medicine “Honglian”, which have good medicinal value and research value. In addition, due to the significant impacts of global warming on the habitat environment of *Lagotis macrosiphon* P. C. Tsoong & Yang (*Lagotis macrosiphon*), *Lagotis brevituba* Maxim. (*Lagotis brevituba*), *Lagotis integra* W. W. Sm. (*Lagotis integra*), *Lagotis angustibracteata* P. C. Tsoong & Yang (*Lagotis angustibracteata*), *Lagotis ramalana* Batalin (*Lagotis ramalana*) and *Lagotis brachystachya* Maxim. (*Lagotis brachystachya*), it becomes necessary to predict the suitable distribution and environmental factors of the *Lagotis* genus. Based on the predicted future suitable distribution area, the specific location for in situ conservation of the genus *Lagotis* can be determined. Second, based on the predicted major influencing environmental factors, it can provide a basis for the introduction of the *Lagotis* genus to domesticate the suitable cultivation conditions. Consequently, in this study, through surveys and herbarium collections, we gathered 769 effective sites of *Lagotis*, combined with 28 environmental factors, and used the MaxEnt model to predict the current and future suitable habitat distribution for six species of the *Lagotis* genus, clarifying the main environmental factors affecting their geographical distribution, so as to provide a scientific basis for the efficient use of *Lagotis* and conservation measures.

## 2 Materials and Methods

### 2.1 Data Sources

#### 2.1.1 Data and Variable Sources

**FIGURE 1:**
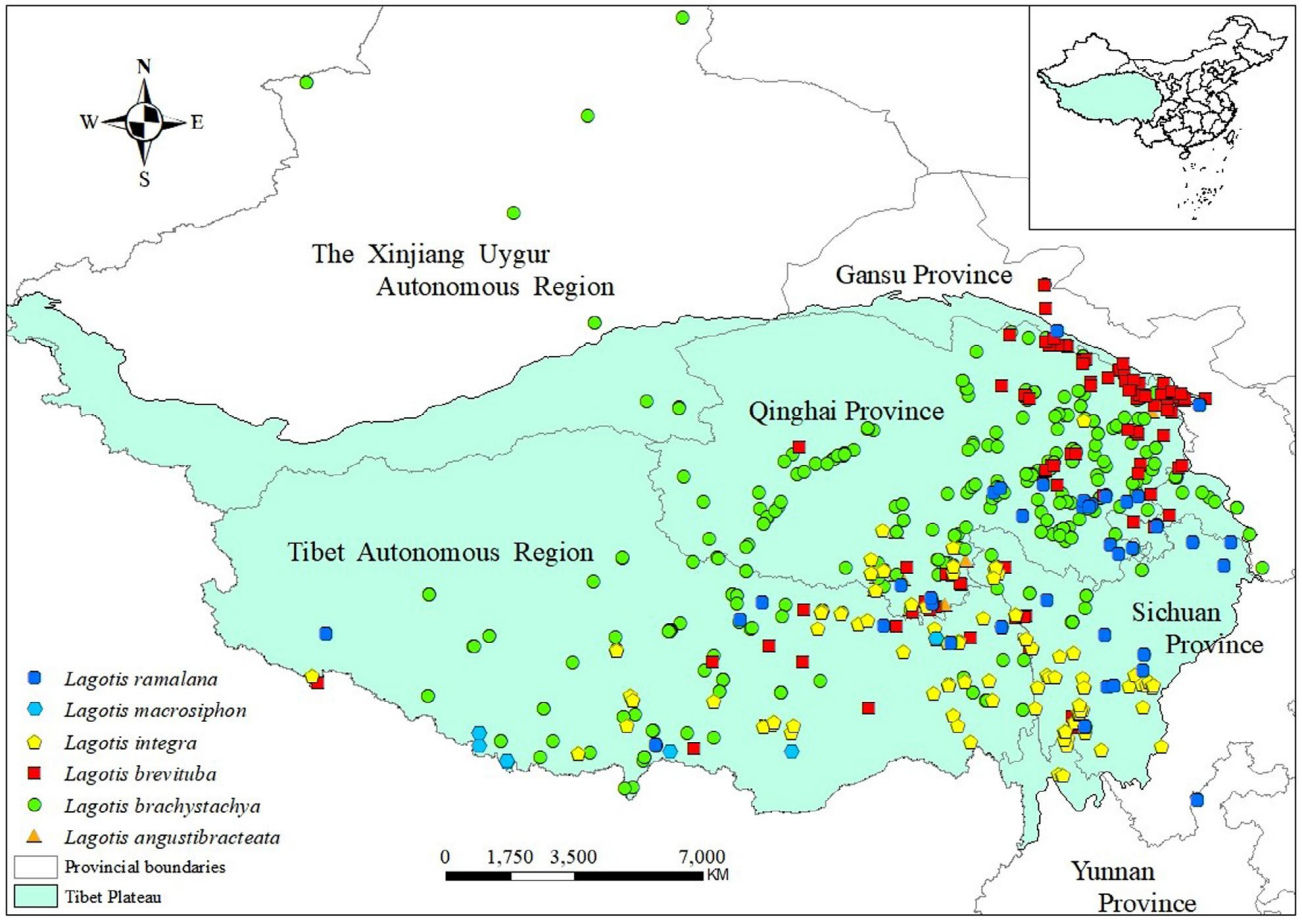
Modern occurrence sites of the six *Lagotis*.

The location data of *Lagotis* came from the field collection survey of the group, and most of the data locations were difficult to collect, so the data were collected through the China Digital Herbarium (http://www.cvh.ac.cn/), NSII China Specimen Resource Platform (http://www.nsii.org.cn/2017/home.php), Global Biodiversity Information Facility (https://www.gbif.org/zh/), and China Plant Image Library (http://ppbc.iplant.cn/) to collect data. Specific latitude and longitude data from various platforms were adopted and collected. For data that lacked accurate latitude and longitude coordinates in the recorded data, we used Google Earth (http://ditu.google.cn/) to determine these coordinates based on the geographic location described (Deng et al. 2014; Shi et al. 2024; Zhang et al. 2023). This allows for a clearer and more comprehensive picture of the contemporary distribution of *Lagotis*. Since the proximity of sample points can cause spatial correlation effects, to minimize this phenomenon, a total of six *Lagotis* species were obtained after eliminating redundant data with duplicate or unclear latitude and longitude information using EMTools software: *Lagotis macrosiphon*, *Lagotis brevituba*, *Lagotis integra*, *Lagotis angustibracteata*, *Lagotis ramalana*, and *Lagotis brachystachya* with effective distribution point data of 17, 115, 152, 18, 46, and 421, respectively (Figure 1). See Appendix S1 for specific data. Subsequently, the latitude and longitude coordinates of the samples were saved in an Excel file and converted into the format required for MaxEnt modeling in the CSV format required for MaxEnt construction (Liao et al. 2023; Zhan et al. 2022).

#### 2.1.2 Data Acquisition and Screening of Environmental Factors

A total of 28 environmental factors were selected for this study (Table 1). Among them, 19 bioclimatic factors (bio1–bio19) and elevation variables (alt) were downloaded from the WorldClim Global Climate Database (version 1.4) (http://www.worldclim.org) at a spatial resolution of 30″(about 1 km^2^), which are the most widely used climatic and topographic variables in the modeling of the potential distribution of species (Remya et al. 2015; Ma and Sun 2018). In addition, eight soil variables were downloaded from the Qinghai‐Xizang Plateau National Data Center (http://data.tpdc.ac.cn/zh‐hans/) (Table 1). In order to maintain the same scale as the bioclimatic factors, the eight soil variables were resampled at a spatial resolution of 30″ (about 1 km^2^) (Zhang et al. 2011).

**TABLE 1:** List of environmental variables used in this research.

Type	Variable	Description (Unit)
Bioclimatic variable	bio1	Annual mean temperature (°C)
	bio2	Monthly diurnal range (mean of monthly (max temp − min temp)) (°C)
	bio3	Isothermality (bio02/bio07) (×100) (°C)
	bio4	Temperature seasonality (standard deviation ×100) (°C)
	bio5	Max temperature of the warmest month (°C)
	bio6	Min temperature of the coldest month (°C)
	bio7	Temperature annual range (Bio5–Bio6) (°C)
	bio8	Mean temperature of wettest quarter (°C)
	bio9	Mean temperature of the driest quarter (°C)
	bio10	Mean temperature of the warmest quarter (°C)
	bio11	Mean temperature of the coldest quarter (°C)
	bio12	Annual precipitation (mm)
	bio13	Precipitation of the wettest month (mm)
	bio14	Precipitation of the driest month (mm)
	bio15	Precipitation seasonality (coefficient of variation)
	bio16	Precipitation of the wettest quarter (mm)
	bio17	Precipitation of the driest quarter (mm)
	bio18	Precipitation of the warmest quarter (mm)
	bio19	Precipitation of the coldest quarter (mm)
	alt	Elevation (m)
Soil variable	ak	Available potassium (mg/kg)
	an	Available nitrogen (mg/kg)
	ap	Available phosphorus (mg/kg)
	pH	pH values
	som	Organic matter (g/kg)
	tk	Total potassium (mg/L)
	tp	Total phosphorus (mg/kg)
	tn	Total nitrogen (mg/kg)

There is a serious problem of multicollinearity among the bioclimatic variables. In order to select the variables with strong predictive ability for the model, we eliminated the multicollinearity among the 28 environmental variables and established the Pearson correlation coefficients (Wen et al. 2016). Using the ArcGIS 10.8 software to extract environmental factor information from the distribution points of the *Lagotis* genus, calculate the Pearson correlation coefficients between the environmental factors, the correlation coefficient was 0.8 as the threshold. For each significantly correlated variable, the exclusion method was used to delete variables with zero contribution, environmental factors with a correlation coefficient > 0.8 should retain the one with the highest contribution rate. And the environmental factors with Pearson correlation coefficients < 0.8 and relatively high contribution were finally retained (Tang et al. 2021). See Appendix S2 for a table of Pearson correlations. Based on the above methodology, environmental factors that are relatively important for the geographical distribution of each *Lagotis* plant genus were screened out (Table 2).

**TABLE 2:** Environment variables used after post‐screening.

Species	Variables
*Lagotis macrosiphon*	bio3, bio4, bio7, bio12, bio14, alt, tp, pH
*Lagotis brevituba*	bio1, bio2, bio3, bio4, bio12, bio15, alt, som, pH, tk, ap, tp, ak
*Lagotis integra*	bio1, bio2, bio3, bio4, bio6, bio13, bio15, bio17, bio18, alt, pH, an, tp, tk, ak, ap
*Lagotis angustibracteata*	bio1, bio2, bio3, bio4, bio11, bio12, bio15, bio18, bio19, alt, tk, som, ak, pH, ap, tp
*Lagotis ramalana*	bio1, bio2, bio3, bio12, bio14, bio19, alt, ap, pH, an, ak, tk, tp
*Lagotis brachystachya*	bio1, bio2, bio3, bio4, bio12, bio15, alt, pH, an, tp, ak, tk, ap

#### 2.1.3 Sources of Data on Future Climate

The future climate data were obtained from the World Climate Data Network (http://www.worldclim.org/), simulated by the Global Climate Model CCSM4, and three representative GHG (Greenhouse Gas) climate scenarios, RCP2.6 (low emission concentration), RCP6.0 (medium emission concentration), and RCP8.5 (high emission concentration), which are typical of the typical concentration scenarios, were selected to build the model. The three GHG climate scenarios, RCP2.6, RCP6.0, and RCP8.5, were modeled to predict the changes in the area of the suitable habitat of *Lagotis* in the next two periods, 2050s and 2070s. The spatial resolution of each future period was 30″ (about 1 km^2^), and all bioclimatic variables were converted to ASC format for MaxEnt analysis (Zhao, Chen, et al. 2021; Zhao, Deng, et al. 2021; Anand et al. 2021).

### 2.2 Research Methodology

#### 2.2.1 MaxEnt Model Construction

Based on the stability of the MaxEnt model, the latitude and longitude coordinate loci of the six *Lagotis* species were imported into the MaxEnt version 3.4.1 software together with the filtered environmental variables (Table 1). Considering that the selected ecological factors contribute differently to the distribution of *Lagotis* and that the correlation between ecological factors reduces the simulation accuracy of the MaxEnt model, it is important to avoid multicollinearity between variables. For each *Lagotis* genus, the distribution point data and ecological factor data were imported into the model, and factors with a contribution rate equal to 0 were directly excluded according to the results of factor contribution rate (Li et al. 2020). The remaining ecological factors were used to predict the distribution of the corresponding *Lagotis* genera. The results of factor screening are shown in Table 2. After the MaxEnt model construction was completed, the settings were set to generate response curves, ROC (the receiver operating characteristic) curves and the knife‐cut method. To evaluate the range of environmental factor suitability with the response curves, to evaluate the model accuracy with the ROC curves and the area under the AUC (area under curve) curve (Wang, Yang, et al. 2019; Wang, Wu, et al. 2019), and to test the weighting of the environmental factors on the suitable growth conditions of *Lagotis* genus with the knife‐cut method (Jia et al. 2024; Parveen et al. 2022). Based on the output of the MaxEnt model, the SDM tool in ArcGIS software was used to classify the suitable areas of *Lagotis* genus into: low suitable area, medium suitable area, and high suitable area. The distribution area of potential suitable areas in *Lagotis* genus was then calculated by the spatial statistics function of ArcGIS software (Soilhi et al. 2022).

#### 2.2.2 Model Parameter Selection Optimization

In order to make adequate predictions, the parameters were set as follows: tick ‘Create response curves’, ‘Make pictures of predictions’, ‘Do jackknife to measure variable importance’, make ‘Output format’ to “Logistic”, and ‘Output file type’ is “asc” (Hosseini et al. 2024). The model was run using the following settings: the convergence threshold was set to 0.00001, the regularization multiplier parameter was set to 1.0, and the contribution of the environmental variables was assessed using the knife‐cut method with the maximum number of iterations set to 5000 (Ab Lah et al. 2021). The species map probability distribution is based on the expression of the gain per iteration, and 5000 iterations are usually chosen to ensure that the model has enough time to optimize its parameters during training to achieve better predictive performance, both to avoid underfitting and to reduce the risk of overfitting to some extent (Boral and Moktan 2021). The model was configured such that 75% of the samples were randomly selected to form the training set and the remaining 25% were designated as the test set (Devi et al. 2024). Set the MaxEnt model to not reset the sampling method, repeat the run parameter settings 10 times, and the average of the 10 runs is used as the result of the ecological suitability calculation and set the other parameters by default (Xu et al. 2020; Mengistu et al. 2023).

#### 2.2.3 MaxEnt Model Evaluation

MaxEnt evaluated the impact of ecological factors on the distribution of the *Lagotis* genus using two methods. The first method is the PC‐PI approach, which includes the percentage contribution (PC) and the permutation importance (PI) (Abdelaal et al. 2019). The percentage contribution (PC) indicates the extent to which each ecological factor contributes to the geographical distribution of *Lagotis* species during the model training process. The permutation importance (PI) measures the decline in AUC value when ecological factors in the training sample points are randomly permuted and the model is re‐run. A larger decrease in AUC value signifies a higher degree of model dependence on that particular variable (Fitzgibbon et al. 2022). The MaxEnt model calculates the AUC value of the area under the ROC curve to evaluate the prediction accuracy of the model, and the value of AUC is from 0 to 1. The AUC value of 0.5–0.6 means that the model fails in prediction; 0.6–0.7 means that the prediction result is poor; 0.7–0.8 means that the prediction result is average; 0.8–0.9 means that the prediction result is good; 0.9–1 means that the prediction result is very good (Puchałka et al. 2023). The other is the Jackknife test was used to quantify the relative significance of each predictor variable in the predictive model (Yang et al. 2013). The knife‐cut method automatically generates a plot of the influence of the predictors on the model during the MaxEnt (Wan et al. 2024). The dark blue bar indicates the amount of gain generated by the unique variable; the longer the bar, the greater the contribution of the variable and the more critical it is to the distribution of the *Lagotis* genus; the light blue bar indicates the amount of gain of all the remaining variables after the removal of the unique variable, and the longer the bar, the less impact it has on the model; and the red bar indicates the cumulative amount of gain of all the predictors (Wang et al. 2023).

### 2.3 Projections of the Distribution Area of the *Lagotis* Genus

A species distribution model was fitted to the current time, and then the fitted model was projected to future climate scenarios (Rana et al. 2017). Then compared the differences in the suitability zones of the *Lagotis* genus in different time periods were compared and a map of the changes in the spatial distribution pattern of the *Lagotis* genus under the future climate change scenarios was obtained (Rawat et al. 2022). The results were used to predict the changes in habitat suitability of *Lagotis* under different climate scenarios (2050s and 2070s).

## 3 Results and Analysis

### 3.1 MaxEnt Model Prediction Accuracy

**FIGURE 2:**
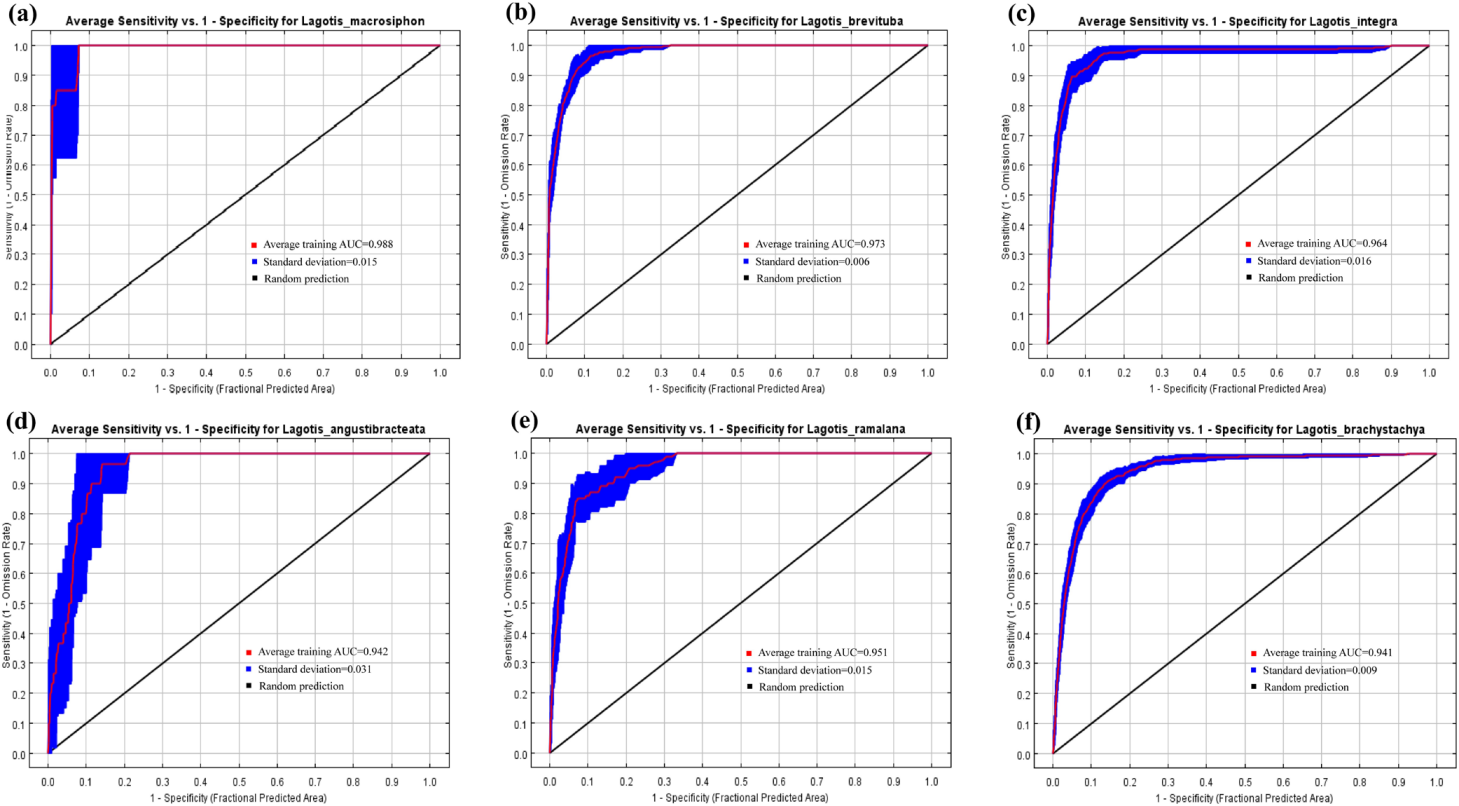
ROC validation curves of predicted results for the distribution of six types of *Lagotis* by MaxEnt model under the current situation. (a) *Lagotis macrosiphon*, (b) *Lagotis brevituba*, (c) *Lagotis integra*, (d) *Lagotis angustibracteata*, (e) *Lagotis ramalana*, (f) *Lagotis brachystachya*.

**FIGURE 3:**
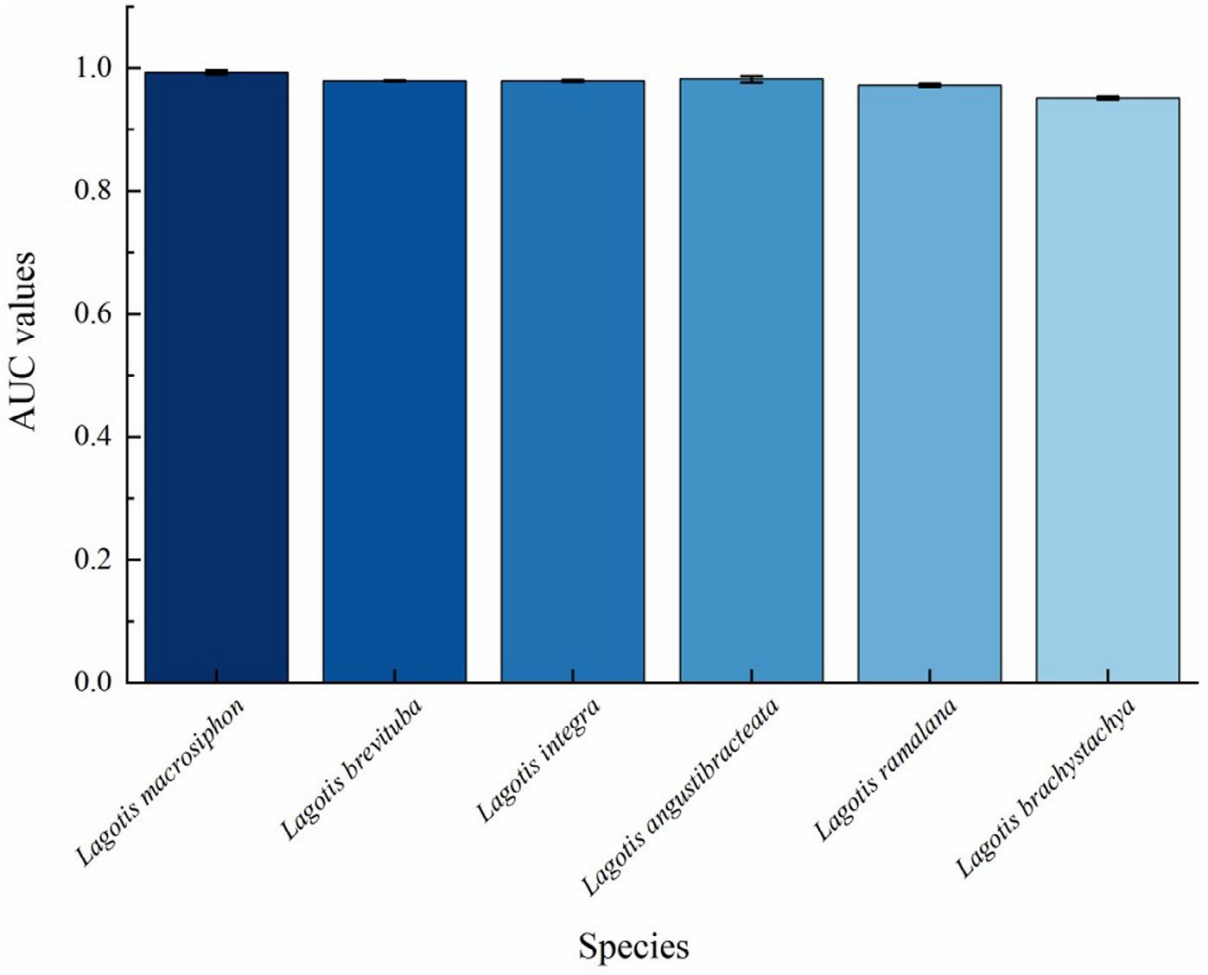
Standard deviations of predicted results for the distribution of six types of *Lagotis* by MaxEnt model under the current situation.

In this study, the AUC values of the training data set of ROC curves were obtained as 0.988, 0.973, 0.964, 0.942, 0.951, and 0.941 of *Lagotis macrosiphon*, *Lagotis brevituba*, *Lagotis integra*, *Lagotis angustibracteata*, *Lagotis ramalana*, *Lagotis brachystachya*. The mean values of random errors are 0.015, 0.006, 0.016, 0.031, 0.015, and 0.009, respectively (Figure 2). The standard deviation of the AUC values after ten repeated runs of the MaxEnt model was small (Figure 3), indicating that the MaxEnt model is accurate and credible in predicting the potential distribution of *Lagotis*.

### 3.2 Screening of Dominant Environmental Factors and Dedication Rate Analysis

**FIGURE 4:**
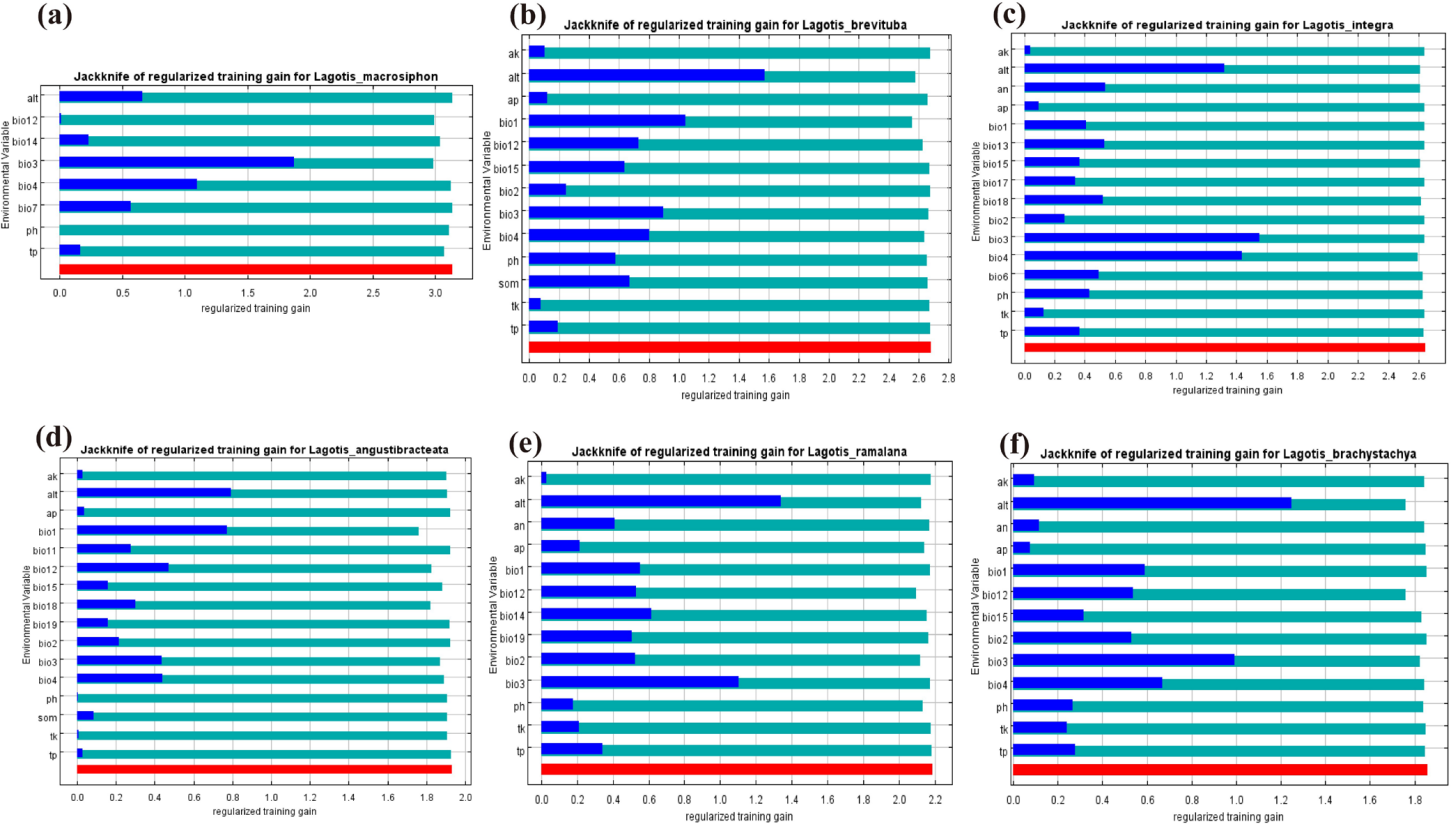
Evaluation of major environmental factors by Jackknife method. (a) *Lagotis macrosiphon*, (b) *Lagotis brevituba*, (c) *Lagotis integra*, (d) *Lagotis angustibracteata*, (e) *Lagotis ramalana*, (f) *Lagotis brachystachya*.

**FIGURE 5:**
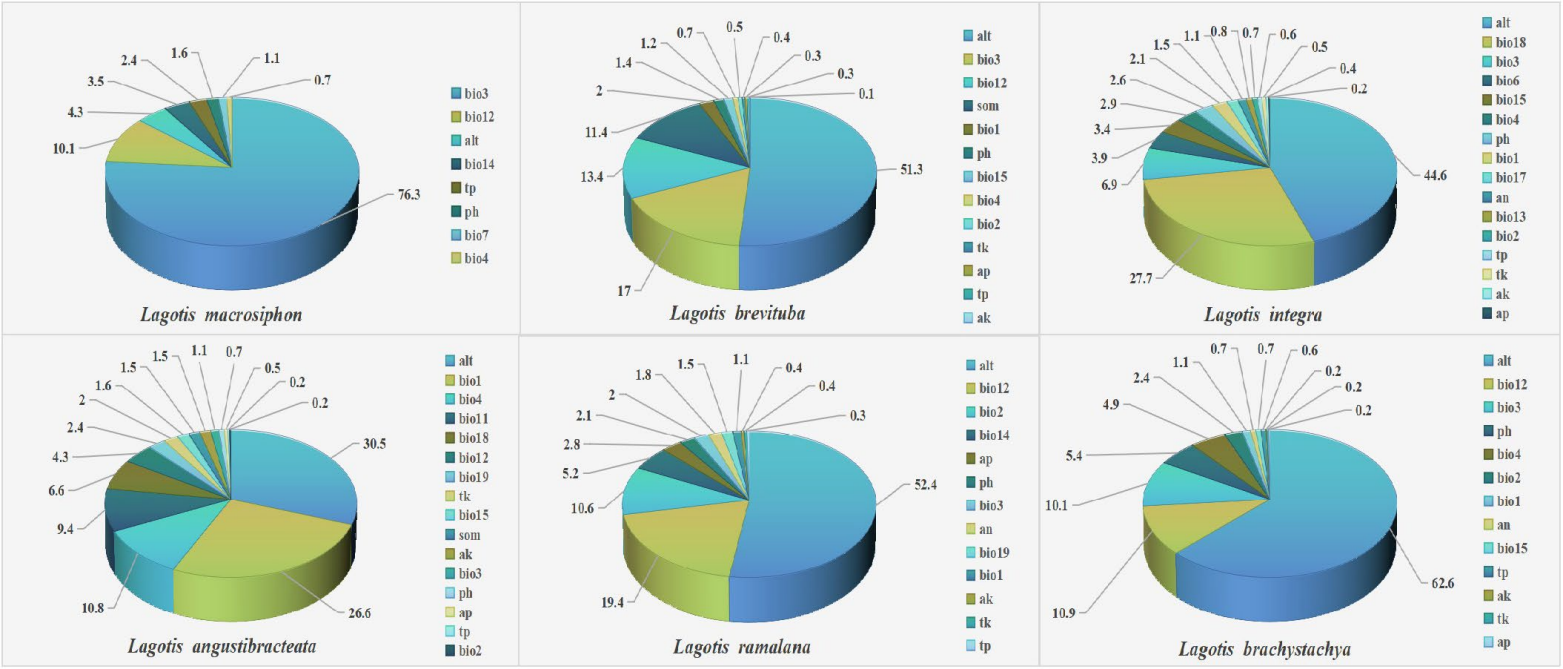
Contribution of environmental factors to the growth and development of six species of *Lagotis*.

The results show that the distribution of *Lagotis* genus plants is mainly influenced by altitude (alt), annual precipitation (bio12) and isothermality (bio3). From Figures 3 and 4, by analyzing the contribution rates of the 19 ecological factors through the MaxEnt model, isothermality (bio3), annual precipitation (bio12), and altitude (alt) have a significant contribution to the distribution of *Lagotis macrosiphon*, *Lagotis_brachystachya*, and *Lagotis_brevituba*, with the combined contribution rate of these three environmental variables reaching 90.7%, 83.6%, and 81.7%. The results show that the three factors—altitude (alt), precipitation of the warmest quarter (bio18) and isothermality (bio3) contributed more to the prediction of the distribution area of *Lagotis integra*, with the combined contribution rate of these three environmental variables reaching 78.7% (Figure 4). Similarly, according to the contribution rate results given by the model, three ecological factors altitude—(alt), annual mean temperature (bio1) and temperature seasonality (bio4) play an important role in the prediction of the distribution area of *Lagotis_angustibracteata*, with the sum of the contribution rates reaching 67.9%. From Figures 4 and 5, it can be seen that the ecological factors altitude—(alt), annual precipitation (bio12) and monthly diurnal range (bio2) have a higher contribution rate to the model's prediction of the distribution of *Lagotis ramalana*, with the sum of the contribution rates reaching 82.45%.

### 3.3 Contemporary Suitable Area Distribution of *Lagotis* Genus

**FIGURE 6:**
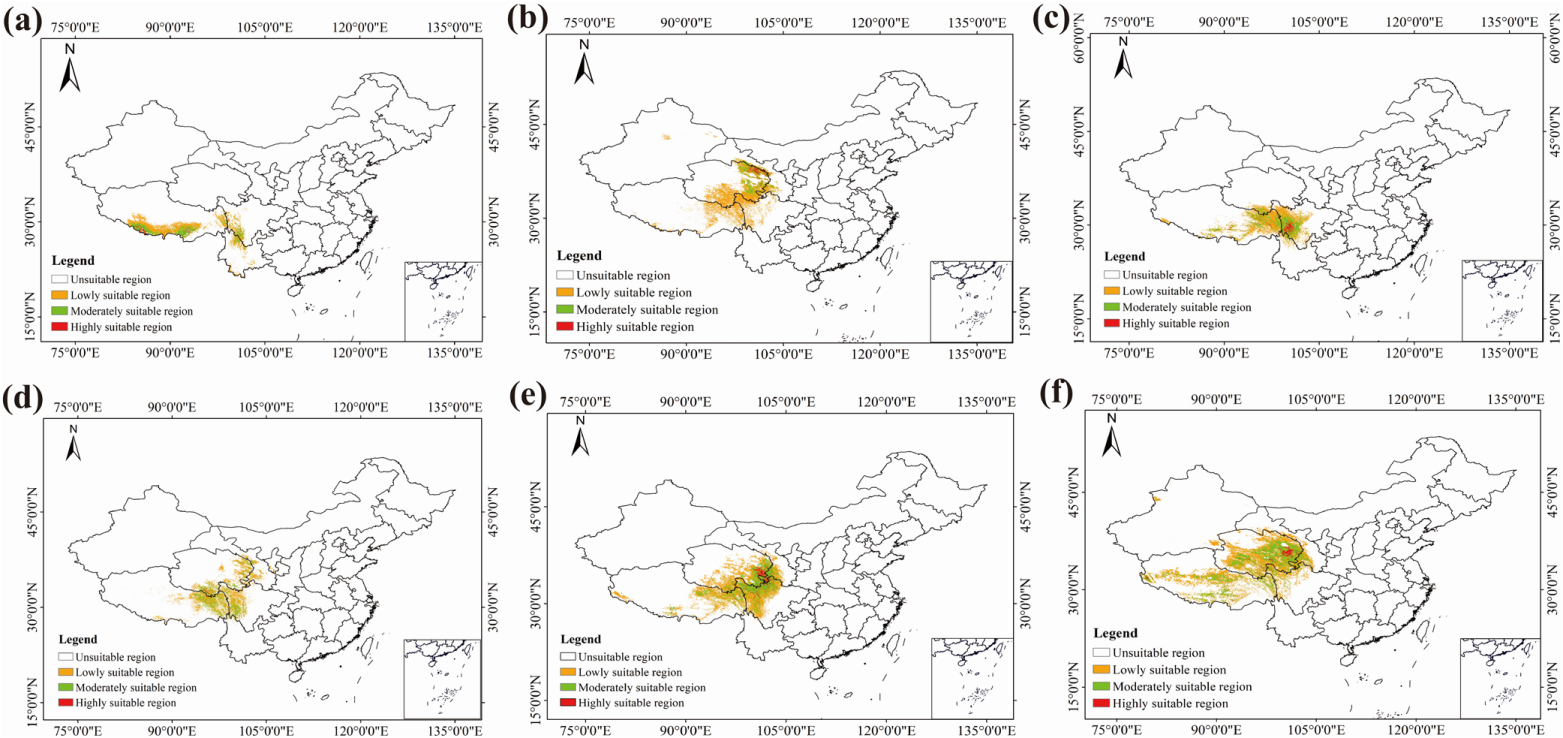
Current distribution of MaxEnt models for six *Lagotis* in China, the color scale from white to red indicates the habitat suitability value from 0 to 1. (a) *Lagotis macrosiphon*, (b) *Lagotis brevituba*, (c) *Lagotis integra*, (d) *Lagotis angustibracteata*, (e) *Lagotis ramalana*, (f) *Lagotis brachystachya*.

In this study, according to the characteristics of *Lagotis* distribution, the ecological suitability derived from MaxEnt was reclassified using ArcGIS software to obtain the ecological suitability zoning map of *Lagotis* (Figure 6). The predictive results indicate that under the current climatic conditions, the ecological suitability distribution area for the *Lagotis* genus is relatively extensive, with a large total area of suitability. The highly suitable areas are primarily located in the eastern, northern, and southeastern parts of Qinghai, the southwestern part of Sichuan, and the southern part of Xizang. The moderately suitable areas are adjacent to the highly suitable areas, mainly in the eastern part of Qinghai, the southern part of Xizang, and the northwestern part of Sichuan. The low suitability areas are interspersed and primarily concentrated in the peripheral regions of the Qinghai‐Xizang Plateau. The total habitat area of *Lagotis macrosiphon* is 29.56 × 10^4^ km^2^. It is divided into a low suitability area spanning 19.78 × 10^4^ km^2^, a moderately suitable habitat area covering 8.56 × 10^4^ km^2^, and a highly suitable area covering 1.26 × 10^4^ km^2^. The habitat is mainly distributed in the southern part of the Qinghai‐Xizang Plateau, including the southwest and southeastern parts of Xizang, northern Yunnan, and southwestern Sichuan (Figure 6). The total suitable habitat area of *Lagotis brevituba* is 49.67 × 10^4^ km^2^. It is divided into a low suitability area spanning 41.35 × 10^4^ km^2^, a moderately suitable habitat area covering 6.60 × 10^4^ km^2^, and a highly suitable area covering 1.72 × 10^4^ km^2^. The habitat is mainly distributed in the northern part of the Qinghai‐Xizang Plateau, including the northeastern and southern parts of Qinghai Province, as well as eastern Xizang and western Sichuan (Figure 6). The suitable habitat area for *Lagotis integra* is 41.66 × 10^4^ km^2^. It is divided into a low suitability area spanning 26.66 × 10^4^ km^2^, a moderately suitable habitat area covering 12.61 × 10^4^ km^2^, and a highly suitable area covering 2.39 × 10^4^ km^2^. This habitat is primarily found in the southeast of the Qinghai‐Xizang Plateau, including eastern Xizang, southern Qinghai, and western Sichuan (Figure 6). The total suitable habitat area of *Lagotis angustibracteata* is 37.92 × 10^4^ km^2^. It is divided into a low suitability area spanning 26.33 × 10^4^ km^2^, a moderately suitable habitat area covering 11.25 × 10^4^ km^2^, and a highly suitable area covering 0.34 × 10^4^ km^2^. The habitat is mainly distributed in the eastern part of the Qinghai‐Xizang Plateau, including the eastern and southern parts of Qinghai Province, as well as eastern Xizang and western Sichuan (Figure 6). The total habitat area of *Lagotis ramalana* is 66.03 × 10^4^ km^2^. It is divided into a low suitability area spanning 41.23 × 10^4^ km^2^, a moderately suitable habitat area covering 20.91 × 10^4^ km^2^, and a highly suitable area covering 3.89 × 10^4^ km^2^. The habitat is mainly distributed in the eastern part of the Qinghai‐Xizang Plateau, including the eastern and southern parts of Qinghai Province, as well as eastern Xizang and western Sichuan (Figure 6). The total suitable habitat area of *Lagotis brachystachya* is 91.05 × 10^4^ km^2^. It is divided into a low suitability area spanning 59.09 × 10^4^ km^2^, a moderately suitable habitat area covering 29.16 × 10^4^ km^2^, and a highly suitable area covering 2.80 × 10^4^ km^2^. The habitat is mainly distributed in the eastern and southern parts of the Qinghai‐Xizang Plateau, including large parts of Qinghai Province, as well as in southern Xizang and northwest Sichuan (Figure 6).

### 3.4 Future Distribution Prediction and Fluctuation Analysis of Suitable Habitats

**FIGURE 7:**
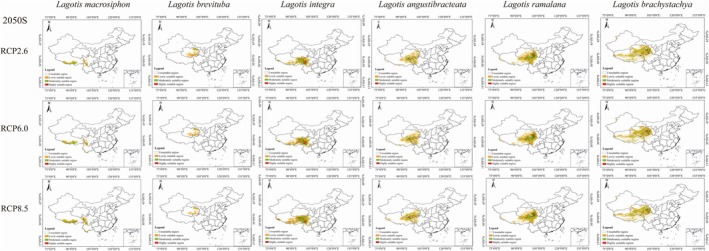
Prediction of the suitable range of the six species of *Lagotis* in 2050.

**FIGURE 8:**
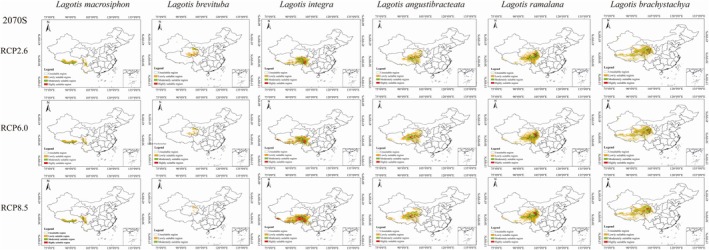
Prediction of the suitable range of the six species of *Lagotis* in 2070.RCP, representative concentration pathway.

**FIGURE 9:**
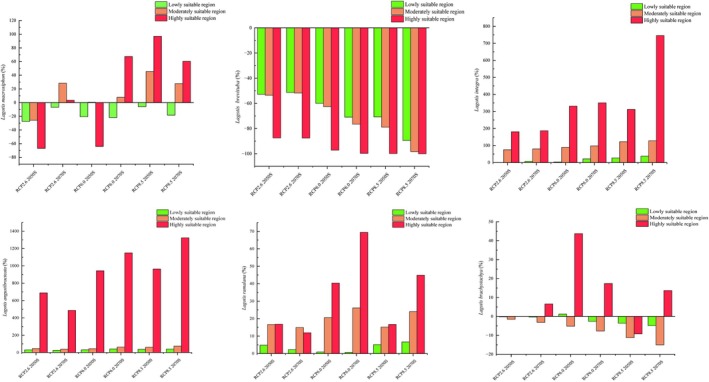
Four different levels of fluctuation in China's future climate scenarios: Green, blue and navy represent the proportion of low, medium and high suitability areas and unsuitable habitats, respectively.

According to the predictions from the MaxEnt model, the potential suitable habitat distribution for *Lagotis* in the 2050s and 2070s is forecasted under three different climate scenarios: RCP2.6, RCP6.0, and RCP8.5. Compared to the current climate conditions, the suitable habitat distribution area is expected to change. The suitable habitat area for *Lagotis integra*, *Lagotis angustibracteata*, and *Lagotis ramalana* are expected to increase with the rise in carbon dioxide concentration, with the area of their highly suitable regions showing a particularly significant increase (Figures 7 and 8). The growth percentage ranges are 180.82% to 795.94%, 688.43% to 1149.45%, and 11.89% to 69.42%, respectively (Figure 9). Under the RCP2.6 model, the area of highly suitable areas for *Lagotis integra* is increasing by 4.31 × 10^4^ km^2^ and 4.46 × 10^4^ km^2^ in 2050 and 2070, respectively. Concurrently, Under the RCP6.0 model, the area of highly suitable regions increased by 7.90 × 10^4^ km^2^ in 2050 and by 8.37 × 10^4^ km^2^ in 2070. Under the RCP8.5 model, the area of highly suitable areas is increasing by 7.45 × 10^4^ km^2^ and 17.79 × 10^4^ km^2^, respectively (Figures 7 and 8). Similarly, Under the RCP2.6 model, the area of highly suitable areas for *Lagotis angustibracteata* is increasing by 2.36 × 10^4^ km^2^ and 1.67 × 10^4^ km^2^ in 2050 and 2070, respectively. Concurrently, Under the RCP6.0 model, the area of highly suitable regions increased by 3.23 × 10^4^ km^2^ in 2050 (Figure 7) and by 3.94 × 10^4^ km^2^ in 2070 (Figure 8). Under the RCP8.5 model, the area of highly suitable areas is increasing by 3.30 × 10^4^ km^2^ and 4.53 × 10^4^ km^2^, respectively. Under the RCP2.6 model, the area of highly suitable areas for *Lagotis ramalana* is increasing by 0.65 × 10^4^ km^2^ and 0.46 × 10^4^ km^2^ in 2050 and 2070, respectively. Concurrently, Under the RCP6.0 model, the area of highly suitable regions increased by 1.57 × 10^4^ km^2^ in 2050 and by 2.70 × 10^4^ km^2^ in 2070 (Figures 7 and 8). Under the RCP8.5 model, the area of highly suitable areas is increasing by 0.65 × 10^4^ km^2^ and 1.75 × 10^4^ km^2^, respectively. In summary, the total suitable habitat area for *Lagotis integra*, *Lagotis angustibracteata*, and *Lagotis ramalana* has increased compared to the present day. The suitable habitats remain primarily concentrated in eastern Qinghai, eastern Tibet, and western Sichuan (Figures 7 and 8). As the time period increases, the area of high suitability for *Lagotis macrosiphon* increases. In the 2070s, the highly suitable areas for *Lagotis macrosiphon* on the Qinghai‐Xizang Plateau have increased to varying degrees, by 3.37%, 67.37%, and 60.32%, respectively; specifically, the highly adapted area increased by 0.04 × 10^4^ km^2^, 0.85 × 10^4^ km^2^ and 0.76 × 10^4^ km^2^, mainly occupying the southern and eastern parts of Xizang, and the western part of Sichuan (Figure 9). In contrast, the suitable habitat area for *Lagotis brevituba* is projected to decrease in all scenarios compared to the present. Under the RCP2.6 scenario, the total suitable area for *Lagotis brevituba* shrinks by 26.90 × 10^4^ km^2^ in 2050 and by 26.17 × 10^4^ km^2^ in 2070, an overall reduction of 54.16% and 52.69%. Under the RCP6.0 scenario, the total suitable area for *Lagotis brevituba* is projected to shrink by 30.61 × 10^4^ km^2^ in 2050 and by 36.12 × 10^4^ km^2^ in 2070, representing an overall reduction of 61.62% and 72.72%, respectively. In parallel, under the RCP8,5 scenario, the total suitable area for *Lagotis brevituba* is projected to shrink by 36.19 × 10^4^ km^2^ in 2050 and by 45.23 × 10^4^ km^2^ in 2070, representing an overall reduction of 72%, 86% and 91.05%, respectively. In conclusion, the area of suitable areas for *Lagotis brevituba* will show a decreasing trend in the future, and the suitable areas are still mainly concentrated in the habitats primarily distributed in the northeast and south of Qinghai Province, as well as in the east of Tibet and the west of Sichuan (Figure 9). For *Lagotis brachystachya*, compared to the present, the area of moderate suitability is gradually decreasing, while the area of high suitability is increasing. Under the RCP2.6 scenario, the moderately suitable area of *Lagotis brachystachya* is projected to decrease by 0.44 × 10^4^ km^2^ in 2050 and by 0.92 × 10^4^ km^2^ in 2070, representing reductions of 1.51% and 3.14%, respectively. In contrast, the area of highly suitable areas increased by 0.18 × 10^4^ km^2^ in 2070, and the growth percentage is 6.59%. Under the RCP6.0 scenario, the moderately suitable area of *Lagotis brachystachya* is projected to decrease by 1.51 × 10^4^ km^2^ in 2050 and by 2.24 × 10^4^ km^2^ in 2070, representing reductions of 1.51% and 3.14%, respectively. In contrast, the area of highly suitable areas increased by 1.22 × 10^4^ km^2^ in 2050 and by 0.49 × 10^4^ km^2^ in 2070; the growth percentages are 43.74% and 17.40%. Under the RCP8.5 scenario, the moderately suitable area of *Lagotis brachystachya* is projected to decrease by 3.27 × 10^4^ km^2^ in 2050 and by 4.38 × 10^4^ km^2^ in 2070, representing reductions of 11.23% and 15.02%, respectively. In contrast, the area of highly suitable areas increased by 0.38 × 10^4^ km^2^ in 2070; the growth percentage is 13.67%. The suitable habitats remain primarily concentrated in the eastern, southern, and central regions of Qinghai (Figure 9).

## 4 Discussion

### 4.1 Main Ecological Factors Dominating the Distribution of *Lagotis*

Based on the MaxEnt model, this study predicts the potential suitable distribution areas for *Lagotis* genus in the Qinghai‐Xizang Plateau region under current and future climate conditions, quantifying the dominant ecological factors affecting the species' potential distribution. The simulation prediction results show that the most significant ecological factor affecting the potential suitable distribution area of *Lagotis* genus is altitude (alt), indicating a strong dependency on altitude. Its most suitable distribution area is between 3500 and 5300 m above sea level, confirming it as a unique medicinal plant adapted to alpine climates, consistent with Tibetan Medicine Glossary (1991) records. Chen et al. (2022) used the MaxEnt and ArcGIS models to predict the potential suitable habitat of *Coelonema draboides* in the Qilian Mountains, and the study results showed that altitude (alt) and the mean diurnal temperature range (bio2) have contribution rates of 33.7% and 19.3%, respectively, indicating that the dominant factors affecting the distribution of *Coelonema draboides* include altitude and the mean diurnal temperature range. Li, Su, et al. (2024), Li, Zhaxi, et al. (2024), Li, Ma, et al. (2024) used the MaxEnt model to predict the potential distribution of *Rhodiola tangutica* in the Qinghai‐Xizang Plateau and drew its suitable habitat distribution map with the help of ArcGIS software, showing that altitude and precipitation in the driest month are the main ecological factors affecting the suitable area distribution of *Rhodiola tangutica*. Zhao, Chen, et al. (2021), Zhao, Deng, et al. (2021) used the MaxEnt model to explore the spatial distribution pattern of the suitable habitat of the Tibetan medicine *Lamiophlomis rotata* (Benth.) Kudo in the Qinghai‐Xizang Plateau region, and the analysis showed that altitude and September precipitation have the greatest impact on the suitability of *Lamiophlomis rotata*. These are consistent with the prediction results of this study, and from these results, it can be seen that the main factor limiting the distribution of species in the Qinghai‐Xizang Plateau is altitude (alt). In contrast, Xu et al. (2009) used the MaxEnt model to simulate the distribution of Qinghai spruce (*Picea crassifolia*) in the Qilian Mountain area and concluded that the max temperature of the warmest month and the mean temperature of the wettest quarter are the dominant environmental factors affecting its distribution. This presentation of results may be due to the scale differences in the predicted distribution range. In this study, our prediction of the potential distribution of *Lagotis* genus is based on the Qinghai‐Xizang Plateau region, which far exceeds the scale of the Qilian Mountains. The study results also show that the climate factor annual precipitation (bio12) has a significant impact on the distribution of *Lagotis* genus, consistent with the results of Hu, Long, et al. (2023), Hu, Wei, et al. (2023) prediction of the potential distribution areas of the endangered plants *Anisodus tanguticus* and *Anisodus luridu* in the Qinghai‐Xizang Plateau. Under the background of climate change, the suitable areas for species in the Qinghai‐Xizang Plateau region are subject to increased evaporation, and thus are more affected by drought and salinity stress, so precipitation has a relatively large impact on the growth of *Anisodus tanguticus* and *Anisodus luridu*. Zhao et al. (2024) used the MaxEnt model to study the response of the suitable habitat of *Hordeum vulgare* var. *coeleste* L. to climate change in the Qinghai‐Xizang Plateau, and the analysis found that the most critical environmental factor affecting the distribution of *Hordeum vulgare* var. *coeleste* L. is annual precipitation (bio12), followed by the mean annual temperature (bio1). Precipitation and temperature affect its yield by meeting the growth and development needs of *Hordeum vulgare* var. *coeleste* L. In this study, from the analysis of precipitation response curve, precipitation can effectively alleviate the growth restriction of *Lagotis* in the high altitude area of the Qinghai‐Xizang Plateau. In addition, isothermality (bio3) also indirectly affects the distribution of *Lagotis*. Combined with the knife cut map and correlation analysis data, *Lagotis* has a strong adaptability to low temperature. There may be a special anti‐freeze mechanism in place. However, in reality, there are many uncertain factors that can affect species distribution, such as soil factors, solar radiation, etc. (Indryani et al. 2011). This study's predictions based on the MaxEnt model did not take other factors into account, so there may be slight differences between the potential distribution areas obtained and the actual distribution areas.

### 4.2 Changes in Suitable Habitat for *Lagotis* on the Qinghai‐Xizang Plateau

This study uses the MaxEnt model to predict the potential suitable distribution areas for *Lagotis* genus in the Qinghai‐Xizang Plateau region under current and future climate scenarios. The results indicate that under future climate change conditions, the *Lagotis* genus will still be mainly distributed in the eastern and southern parts of Qinghai, specifically in high‐altitude areas such as Qilian, Gonghe, Xinghai, and Yushu in Qinghai Province, and also in other high‐altitude areas in southern and eastern Xizang, and western Sichuan, which is basically consistent with the known distribution of *Lagotis* genus (Yuan et al. 2016). Analysis of the fluctuation of the suitable habitat area of *Lagotis* genus under future climate scenarios reveals that with global warming and the passage of time, the areas of medium and high suitability for *Lagotis integra*, *Lagotis angustibracteata*, and *Lagotis ramalana* are gradually increasing, but the suitable region is still in the central and southern parts of the Qinghai‐Xizang Plateau. *Lagotis macrosiphon* also shows an increasing trend in highly suitable areas over time. Yang et al. (2024) used the MaxEnt model to predict the potential suitable area of 12 threatened medicinal plants in the Qinghai‐Xizang Plateau. The results showed that 33.3% of the threatened medicinal plants showed an increase in their future habitat area because of their physiological characteristics, which are more adaptable to a wide range of climates. The climatic suitable habitat for 50% of the threatened medicinal plants would migrate to higher altitudes or higher latitude regions. Li et al. (2019) studied the future distribution of *Picea likiangensis* and *Picea purpurea* in the Qinghai‐Xizang Plateau under climate change scenarios and found that under the background of future climate warming, it is conducive to the expansion of *Picea purpurea*. This is similar to the changes in the suitable areas for *Lagotis* genus plants discussed in this study. The suitable area for *Lagotis brevituba* shows a decreasing trend compared to the present, especially in the medium and high suitability areas. The area of moderate suitability for *Lagotis brachystachya* is decreasing, while the area of high suitability is increasing. Therefore, under the conditions of climate change that global warming has a significant impact on the suitable habitat area of *Lagotis* genus.

### 4.3 Response Measures and Issues

The Qinghai‐Xizang Plateau has rich medicinal plant resources; *Lagotis*, a medicinal plant studied in this article, is one of them (Ni et al. 2013; Wu et al. 2024; Hu, Long, et al. 2023; Hu, Wei, et al. 2023). The *Lagotis* genus is one of the important medicinal plant species in the raw materials of Tibetan medicine. Qinghai Province is the main distribution area for this species and also the most important authentic production area for this resource species (Zhang et al. 2020). The vast suitable distribution area in Qinghai Province has created a unique environmental condition for the large‐scale breeding of *Lagotis* resources, and the resource reserves are also significantly higher than those in other distribution areas in the country. Although *Lagotis* resources are currently mainly limited to use as Tibetan medicinal materials, and the actual resource usage is relatively low, with further in‐depth research on the properties of this resource species and further understanding of its potential value, new product types may be developed, and there is a great prospect for development and utilization (Cao et al. 2015). Based on this, this study suggests the following protective measures: (1) The *Lagotis* genus mainly grows in the high mountain shady slope gravel areas, and the living environment is quite special, showing great vulnerability in resource survival. It is necessary to establish protection bases for wild resources as soon as possible and strengthen the protection of resources. Based on the ecological distribution data, highly suitable areas for the *Lagotis* plant can be identified, which are usually favorable for the growth and reproduction of *Lagotis*. Nature reserves should be established or expanding nature reserves in areas with a high concentration of *Lagotis* plants, such as the Qilian Mountains and the Hengduan Mountains. These reserves should cover the main habitats of the *Lagotis* plant, including alpine meadows and hillside meadows, to provide a relatively stable living environment for the plant and reduce the interference of human activities. Focused monitoring should be carried out in the established nature reserves, and regular surveys should be conducted on the population size, growth status, and habitat changes of the *Lagotis* plant. Once it is found that the habitat is threatened or the population is declining, timely protective measures will be taken, such as strengthening patrols and prohibiting digging. At the same time, highly suitable areas can be included in the scope of the red line of ecological protection, strictly limiting development and construction activities to ensure the integrity and stability of their ecosystems. At the same time, according to the biological characteristics of the *Lagotis* genus, determine the most suitable harvest period to improve the quality and yield of medicinal materials, reduce damage to resources and quality, and promote the realization of sustainable utilization goals. (2) It is very difficult to cultivate or propagate resources in the original environment. At present, not only has an effective artificial breeding technology system not been established, but relevant research work has not yet been involved. With the increase in the use of the *Lagotis* genus, excessive mining will lead to a sharp decrease in wild resource reserves and the deterioration of the ecological environment. Strengthen preliminary research on artificial breeding or wild propagation of resources, and quickly study and solve key technical issues in the artificial cultivation or auxiliary breeding of *Lagotis* resources. (3) At present, the research on the effective components and pharmacological effects of the *Lagotis* genus is still in the initial stage, and most of the active components and practical effects in the resources are not very clear. For this reason, a comprehensive and systematic in‐depth study of the chemical components of the *Lagotis* genus, pharmacological activity screening, practical effects, and other aspects should be carried out to explore the potential value of resources. Through relevant research, it can also avoid the blindness in the development and utilization of resources, and is of great significance for giving full play to the value of resource utilization. Although *Lagotis* is not included in the national key protection list, its ecological value and protection needs should not be ignored. It is recommended that local governments include them in the local protection list according to the actual situation and formulate relevant protection regulations to clarify protection measures and responsibilities.

## 5 Conclusions

This study simulated the potential suitable habitat distribution of *Lagotis* genus in the Qinghai‐Xizang Plateau under future climate change scenarios (three RCP scenarios) based on the MaxEnt model and explored the main environmental factors affecting its distribution. The results show that altitude (alt), annual precipitation (bio12), and temperature seasonality (bio3) are the main environmental factors affecting the distribution of suitable areas for *Lagotis* genus. It is inferred that *Lagotis* genus is suitable for growing in high mountain talus slopes, nearby meadows, and high mountain summit stony and cold desert areas. It also has strong adaptability to low temperatures and may have a special antifreeze mechanism, making it an ideal material for studying plant antifreeze mechanisms and screening antifreeze genes. Under the current climate background, the potential suitable areas for *Lagotis* genus are mainly in the eastern, southern, and central parts of the Qinghai‐Xizang Plateau, with no significant trend of change. Therefore, according to the distribution location of its habitat area, the southeastern and central part of the Qinghai‐Xizang Plateau can be used as an important protected area for in situ conservation of *Lagotis* genus in the future. Nature reserves or eco‐protected areas should also be established to ensure the safe reproduction of *Lagotis* in its native environment. This study reveals the potential suitable distribution areas of *Lagotis* genus in the Qinghai‐Xizang Plateau region under current and future climate backgrounds, providing a reference for the future conservation and utilization of this species.

## Author Contributions

**Huiyuan Ma:** conceptualization (lead), formal analysis (lead), investigation (lead), software (lead), visualization (lead), writing – original draft (lead). **Bo Wang:** conceptualization (supporting), investigation (supporting), methodology (supporting), resources (lead), software (supporting), writing – review and editing (lead). **Xue Yang:** investigation (supporting), methodology (supporting), validation (supporting). **Guoying Zhou:** conceptualization (lead), funding acquisition (lead), methodology (supporting), supervision (supporting), writing – review and editing (supporting).

## Conflicts of Interest

The authors declare no conflicts of interest.

## Supporting information


Appendix S1.

Appendix S2.

## Data Availability

All data are in the main text or the Supporting Information.

## References

[ece371319-bib-0001] Ab Lah, N. Z. , Z. Yusop , M. Hashim , J. Mohd Salim , and S. Numata . 2021. “Predicting the Habitat Suitability of *Melaleuca cajuputi* Based on the MaxEnt Species Distribution Model.” Forests 12, no. 11: 1449. 10.3390/f12111449.

[ece371319-bib-0002] Abdelaal, M. , M. Fois , G. Fenu , and G. Bacchetta . 2019. “Using MaxEnt Modeling to Predict the Potential Distribution of the Endemic Plant *Rosa arabica* Crép. In Egypt.” Ecological Informatics 50: 68–75. 10.1016/j.ecoinf.2019.01.003.

[ece371319-bib-0003] Ahmadi, M. , M.‐R. Hemami , M. Kaboli , and F. Shabani . 2023. “MaxEnt Brings Comparable Results When the Input Data Are Being Completed; Model Parameterization of Four Species Distribution Models.” Ecology and Evolution 13, no. 2: 9827. 10.1002/ece3.9827. PMC9937880 36820245

[ece371319-bib-0004] Alami, M. M. , S. Guo , Z. Mei , G. Yang , and X. Wang . 2024. “Environmental Factors on Secondary Metabolism in Medicinal Plants: Exploring Accelerating Factors.” Medicinal Plant Biology 3, no. 1: e016. 10.48130/mpb-0024-0016.

[ece371319-bib-0005] Anand, V. , B. Oinam , and I. H. Singh . 2021. “Predicting the Current and Future Potential Spatial Distribution of Endangered *Rucervus eldii eldii* (Sangai) Using MaxEnt Model.” Environmental Monitoring and Assessment 193, no. 3: 147. 10.1007/s10661-021-08950-1. 33638015

[ece371319-bib-0006] Boral, D. , and S. Moktan . 2021. “Predictive Distribution Modeling of *Swertia bimaculata* in Darjeeling‐Sikkim Eastern Himalaya Using MaxEnt: Current and Future Scenarios.” Ecological Processes 10, no. 1: 26. 10.1186/s13717-021-00294-5.

[ece371319-bib-0007] Cao, L. , Z. Mu , W. Zhong , et al. 2015. “Analysis of Varieties and Standards of Scrophulariaceae Plants Used in Tibetan Medicine.” China Journal of Chinese Materia Medica 40, no. 23: 4686–4692. 10.4268/cjcmm20152327. 27141684

[ece371319-bib-0008] Chen, K. , B. Wang , C. Chen , et al. 2022. “Distribution Prediction and Influential Factors Analysis of Coelonema Draboides, an Endemic and Endangered Plant in Qilian Mountains.” Acta Botanica Boreali‐Occidentalia Sinica 42, no. 11: 1954–1961. 10.7606/j.issn.1000-4025.2022.11.1954.

[ece371319-bib-0009] Deng, F. , X. Li , H. Wang , et al. 2014. “The Suitability of Geographic Distribution and the Dominant Factors of Alfalfa Based on MaxEnt Model in Xilin Gol.” Pratacultural Science 8, no. 10: 1840–1847. 10.11829/j.issn.1001-0629.2013-0686.

[ece371319-bib-0010] Devi, V. , M. H. Fulekar , B. Charles , C. S. Reddy , and B. Pathak . 2024. “Predicting the Habitat Suitability and Species Richness of Plants of Great Himalayan National Park Under Different Climate Change Scenarios.” Environmental Monitoring and Assessment 196, no. 11: 1136. 10.1007/s10661-024-13290-x. 39477861

[ece371319-bib-0011] Dong, R. , B. Chu , R. Hua , et al. 2022. “Geographic Distribution of *Stellera chamaejasme* on the Tibetan Plateau Under Future Climate Scenarios.” Chinese Journal of Grassland 44, no. 4: 10–20. 10.16742/j.zgcdxb.20210375.

[ece371319-bib-0012] Fan, C. , Y. Zhang , S. Pu , et al. 2021. “Complete Chloroplast Genome Sequences of *Lagotis brevituba* (Plantaginaceae): A Famous Tibetan Medicine Plant.” Mitochondrial DNA Part B Resources 6, no. 5: 1638–1639. 10.1080/23802359.2021.1927216. 34104723 PMC8143624

[ece371319-bib-0013] Fitzgibbon, A. , D. Pisut , and D. Fleisher . 2022. “Evaluation of Maximum Entropy (Maxent) Machine Learning Model to Assess Relationships Between Climate and Corn Suitability.” Land 11, no. 9: 1382. 10.3390/land11091382.

[ece371319-bib-0014] Gong, R. , W. Cao , H. Huang , et al. 2022. “Antitumor Potential and Structure Characterization of Polysaccharides From *Lagotis brevituba* Maxim in the Tibetan Plateau.” Frontiers in Nutrition 9: 921892. 10.3389/fnut.2022.921892. 35903443 PMC9320327

[ece371319-bib-0015] Hosseini, N. , H. Mostafavi , and S. M. M. Sadeghi . 2024. “Impact of Climate Change on the Future Distribution of Three *Ferulago* Species in Iran Using the MaxEnt Model.” Integrated Environmental Assessment and Management 20, no. 4: 1046–1059. 10.1002/ieam.4898. 38334016

[ece371319-bib-0016] Hu, D. , X. Long , T. Luobu , and Q. Wang . 2023. “Current Status of Research on Endophytes of Traditional Tibetan Medicinal Plant and Their Metabolites.” 3 Biotech 13, no. 10: 338. 10.1007/s13205-023-03720-x. PMC10495306 37705864

[ece371319-bib-0017] Hu, H. , Y. Wei , W. Wang , et al. 2020. “Richness and Distribution of Endangered Orchid Species Under Different Climate Scenarios on the Qinghai‐Tibetan Plateau.” Frontiers in Plant Science 13: 948189. 10.3389/fpls.2022.948189. PMC9490128 36160966

[ece371319-bib-0018] Hu, H. , Y. Wei , W. Wang , et al. 2023. “Impacts of Climate Change on the Potential Distribution of Endangered Plants *Anisodus tanguticus* and *Anisodus luridus* in the Qinghai‐Tibetan Plateau.” Journal of Lanzhou University (Natural Sciences) 59, no. 2: 182–189. 10.13885/i.issn.0455-2059.2023.02.006.

[ece371319-bib-0019] Indryani, S. , E. Arisoesilaningsih , T. Wardiyati , et al. 2011. “A Model of Relationship Between Climate and Soil Factors Related to Oxalate Content in Porang (*Amorphophallus muelleri* Blume) Corm.” Biodiversitas 12, no. 1: 45–51. 10.13057/biodiv/d120109.

[ece371319-bib-0020] Jia, L. , M. Sun , M. He , M. Yang , M. Zhang , and H. Yu . 2024. “Study on the Change of Global Ecological Distribution of *Nicotiana tabacum* L. Based on MaxEnt Model.” Frontiers in Plant Science 15: 1371998. 10.3389/fpls.2024.1371998. 39091317 PMC11292735

[ece371319-bib-0021] Kaky, E. , and F. Gilbert . 2016. “Using Species Distribution Models to Assess the Importance of Egypt's Protected Areas for the Conservation of Medicinal Plants.” Journal of Arid Environments 135: 140–146. 10.1016/j.jaridenv.2016.09.001.

[ece371319-bib-0022] Kumar, P. 2012. “Assessment of Impact of Climate Change on Rhododendrons in Sikkim Himalayas Using Maxent Modelling: Limitations and Challenges.” Biodiversity and Conservation 21, no. 5: 1251–1266. 10.1007/s10531-012-0279-1.

[ece371319-bib-0023] Lakey, D. K. 2016. “Ecological Status of High Altitude Medicinal Plants and Their Sustainability: Lingshi, Bhutan.” BMC Ecology 16, no. 1: 45. 10.1186/s12898-016-0100-1. 27729077 PMC5059966

[ece371319-bib-0024] Lei, J. , and H. Xu . 2010. “Maxent‐Based Prediction of Potential Distribution of *Solidago canadensis* in China.” Journal of Ecology and Rural Environment 26, no. 2: 137–141.

[ece371319-bib-0025] Li, G. , C. Kim , H. Zha , et al. 2014. “Molecular Phylogeny and Biogeography of the Arctic‐Alpine Genus *Lagotis* (Plantaginaceae).” Systematics and Phylogenys 63, no. 1: 103–115. 10.12705/631.47.

[ece371319-bib-0026] Li, N. , A. Zhang , L. Zhang , et al. 2019. “Predicting Potential Distribution of Two Species of Spruce in Qinghai‐Tibet Plateau Under Climate Change.” Bulletin of Botanical Research 39, no. 3: 395–406. 10.7525/j.issn.1673-5102.2019.03.010.

[ece371319-bib-0027] Li, X. , X. Su , D. Wang , et al. 2024. “Prediction of the Geographical Distribution Pattern of *Rhodiola tangutica* (Crassulaceae) Under the Background of Climate Change, an Endemic Species From the Qinghai‐Tibet Plateau.” Bulletin of Botanical Research 44, no. 2: 168–179. 10.7525/j.issn.1673-5102.2024.02.002.

[ece371319-bib-0028] Li, Y. , M. Li , C. Li , and Z. Liu . 2020. “Optimized Maxent Model Predictions of Climate Change Impacts on the Suitable Distribution of *Cunninghamia lanceolata* in China.” Forests 11, no. 3: 302. 10.3390/f11030302.

[ece371319-bib-0029] Li, Y. , D. Zhaxi , L. Yuan , et al. 2024. “The Effects of Climate Change on the Distribution Pattern of Species Richness of Endemic Wetland Plants in the Qinghai‐Tibet Plateau.” Plants 13, no. 14: 1886. 10.3390/plants13141886. 39065412 PMC11281189

[ece371319-bib-0030] Li, Z. , Y. Ma , Y. Li , et al. 2024. “Spatial and Temporal Variations of the Potential Habitat of *Asterothamnus centraliasiaticus* on the Qinghai‐Tibet Plateau Under Climate Change.” Chinese Journal of Ecology 43, no. 6: 1566–1575. 10.13291/j.1000-4890.202406.025.

[ece371319-bib-0031] Liao, J. , C. Yang , Q. Shao , Q. Sun , and Y. Han . 2023. “Construction of an Ecological Model of *Sambucus javanica* Blume in China Under Different Climate Scenarios Based on Maxent Model.” Plant Ecology 224, no. 3: 221–237. 10.1007/s11258-023-01291-8.

[ece371319-bib-0032] Liu, J. , J. Li , and Y. Lai . 2021. “Plant Diversity and Ecology on the Qinghai‐Tibet Plateau.” Journal of Systematics and Evolution 59, no. 6: 1139–1141. 10.1111/jse.12813.

[ece371319-bib-0033] Liu, Y. , Z. Dao , C. Yang , and C. Long . 2009. “Medicinal Plants Used by Tibetans in Shangri‐la, Yunnan, China.” Journal of Ethnobiology and Ethnomedicine 5, no. 1: 15. 10.1186/1746-4269-5-15. 19416515 PMC2684741

[ece371319-bib-0034] Ma, B. , and J. Sun . 2018. “Predicting the Distribution of *Stipa purpurea* Across the Tibetan Plateau via the MaxEnt Model.” BMC Ecology 18, no. 1: 10. 10.1186/s12898-018-0165-0. 29466976 PMC5822641

[ece371319-bib-0035] Matesanz, S. , and F. Valladares . 2014. “Ecological and Evolutionary Responses of Mediterranean Plants to Global Change.” Environmental and Experimental Botany 103, no. 5: 53–67. 10.1016/j.envexpbot.2013.09.004.

[ece371319-bib-0036] Mengistu, A. G. , W. A. Tesfuhuney , Y. E. Woyessa , and A. S. Steyn . 2023. “Potential Distribution of Selected Invasive Alien Plants Under Current and Future Climate Change Scenarios in South Africa.” Heliyon 9, no. 9: e19867. 10.1016/j.heliyon.2023.e19867. 37809438 PMC10559257

[ece371319-bib-0037] Merow, C. , M. J. Smith , and J. A. Silander Jr . 2013. “A Practical Guide to MaxEnt for Modeling Species' Distributions: What It Does, and Why Inputs and Settings Matter.” Ecography 36, no. 10: 1058–1069. 10.1111/j.1600-0587.2013.07872.x.

[ece371319-bib-0038] Ni, W. , T. Gao , H. Wang , et al. 2013. “Anti‐Fatigue Activity of Polysaccharides From the Fruits of Four Tibetan Plateau Indigenous Medicinal Plants.” Journal of Ethnopharmacology 150, no. 2: 529–535. 10.1016/j.jep.2013.08.055. 24036063

[ece371319-bib-0039] Nippert, J. , P. Fay , J. Carlisle , et al. 2009. “Ecophysiological Responses of Two Dominant Grasses to Altered Temperature and Precipitation Regimes.” Acta Oecologica 35, no. 3: 400–408. 10.1016/j.actao.2009.01.010.

[ece371319-bib-0040] Northwest Institute of Plateau Biology, Chinese Academy of Sciences . 1991. Tibetan Medicine Glossary. Qinghai People's Publishing House.

[ece371319-bib-0041] Parveen, S. , S. Kaur , R. Baishya , and S. Goel . 2022. “Predicting the Potential Suitable Habitats of Genus *Nymphaea* in India Using MaxEnt Modeling.” Environmental Monitoring and Assessment 194, no. 12: 853. 10.1007/s10661-022-10524-8. 36203117

[ece371319-bib-0042] Phillips, S. , and M. Dudík . 2008. “Modeling of Species Distributions With MAXENT: New Extensions and a Comprehensive Evaluation.” Ecography 31, no. 2: 161–175. 10.1111/j.0906-7590.2008.5203.x.

[ece371319-bib-0043] Puchałka, R. , S. Paź‐Dyderska , B. Woziwoda , and M. K. Dyderski . 2023. “Climate Change Will Cause Climatic Niche Contraction of *Vaccinium myrtillus* L. and *V. vitis‐idaea* L. in Europe.” Science of the Total Environment 892: 164483. 10.1016/j.scitotenv.2023.164483. 37268126

[ece371319-bib-0044] Radosavljević, A. , and R. Anderson . 2014. “Making Better Maxent Models of Species Distributions: Complexity, Overfitting and Evaluation.” Journal of Biogeography 41, no. 4: 629–643. 10.1111/jbi.12227.

[ece371319-bib-0045] Rana, S. K. , H. K. Rana , S. K. Ghimire , K. K. Shrestha , and S. Ranjitkar . 2017. “Predicting the Impact of Climate Change on the Distribution of Two Threatened Himalayan Medicinal Plants of Liliaceae in Nepal.” Journal of Mountain Science 14, no. 3: 558–570. 10.1007/s11629-015-3822-1.

[ece371319-bib-0046] Rawat, N. , S. Purohit , V. Painuly , G. S. Negi , and M. P. S. Bisht . 2022. “Habitat Distribution Modeling of Endangered Medicinal Plant *Picrorhiza kurroa* (Royle ex Benth) Under Climate Change Scenarios in Uttarakhand Himalaya, India.” Ecological Informatics 68: 101550. 10.1016/j.ecoinf.2021.101550.

[ece371319-bib-0047] Remya, K. , A. Ramachandran , and S. Jayakumar . 2015. “Predicting the Current and Future Suitable Habitat Distribution of *Myristica dactyloides* Gaertn. Using MaxEnt Model in the Eastern Ghats, India.” Ecological Engineering 82: 184–188. 10.1016/j.ecoleng.2015.04.053.

[ece371319-bib-0048] Renner, I. W. , and D. I. Warton . 2013. “Equivalence of MAXENT and Poisson Point Process Models for Species Distribution Modeling in Ecology.” Biometrics 69, no. 1: 274–281. 10.1111/j.1541-0420.2012.01824.x. 23379623

[ece371319-bib-0049] She, Y. , H. Zhou , Z. Zhang , et al. 2021. “Suitable Distribution of *Notopterygium incisum* in the Three Rivers Headwater Region Under Climate Change.” Ecology and Environmental Sciences 30, no. 10: 2033–2041. 10.16258/j.cnki.1674-5906.2021.10.010.

[ece371319-bib-0050] Shi, J. , M. Xia , G. He , et al. 2024. “Predicting *Quercus gilva* Distribution Dynamics and Its Response to Climate Change Induced by GHGs Emission Through MaxEnt Modeling.” Journal of Environmental Management 357: 120841. 10.1016/j.jenvman.2024.120841. 38581898

[ece371319-bib-0051] Soilhi, Z. , N. Sayari , N. Benalouache , and M. Mekki . 2022. “Predicting Current and Future Distributions of *Mentha pulegium* L. in Tunisia Under Climate Change Conditions, Using the MaxEnt Model.” Ecological Informatics 68: 101533. 10.1016/j.ecoinf.2021.101533.

[ece371319-bib-0052] Sun, T. , B. Deng , Z. Liu , et al. 2011. “Responses of Biomass Allocation of *Lagotis brachystachya* to Degradation of Soil Nutrition Bank in Alpine Meadow.” Pratacultural Science 28, no. 11: 1982–1986.

[ece371319-bib-0053] Suo, X. , C. Liu , Y. Zhao , et al. 2024. “Study on Quality Regionalization of *Forsythia suspensa* (Thunb.)Vah1 in Shanxi Province Based on MaxEnt Model and ArcGIS.” Chinese Journal of Information on Traditional Chinese Medicine 31, no. 10: 1–7. 10.19879/j.cnki.1005-5304.202404180.

[ece371319-bib-0054] Tang, Y. , R. Zhao , G. Ren , et al. 2021. “Prediction of Potential Distribution of *Lycium chinense* Based on MaxEnt Model and Analysis of Its Important Influencing Factors.” Journal of Beijing Forestry University 43, no. 6: 23–32. 10.12171/j.1000-1522.20200103.

[ece371319-bib-0055] Tao, J. , Y. Zhang , J. Dong , et al. 2014. “Elevation‐Dependent Relationships Between Climate Change and Grassland Vegetation Variation Across the Qinghai‐Xizang Plateau.” International Journal of Climatology 35, no. 7: 1638–1647. 10.1002/joc.4082.

[ece371319-bib-0056] Thuiller, W. 2014. “Editorial Commentary on ‘BIOMOD—Optimizing Predictions of Species Distributions and Projecting Potential Future Shifts Under Global Change’.” Global Change Biology 20, no. 12: 3591–3592. 10.1111/gcb.12728. 25200636 PMC4340559

[ece371319-bib-0057] Wan, G. , Q. Li , L. Jin , G. Z. Wan , Q. Q. Li , and J. Chen . 2024. “Integrated Approach to Predicting Habitat Suitability and Evaluating Quality Variations of *Notopterygium franchetii* Under Climate Change.” Scientific Reports 14, no. 1: 26927. 10.1038/s41598-024-77824-6. 39505945 PMC11541729

[ece371319-bib-0058] Wang, R. , H. Yang , W. Luo , et al. 2019. “Predicting the Potential Distribution of the Asian Citrus Psyllid, *Diaphorina citri* (Kuwayama), in China Using the MaxEnt Model.” PeerJ 7, no. 6: e7323. 10.7717/peerj.7323. 31341749 PMC6637924

[ece371319-bib-0059] Wang, T. , T. Wu , P. Wang , R. Li , C. Xie , and D. Zou . 2019. “Spatial Distribution and Changes of Permafrost on the Qinghai‐Tibet Plateau Revealed by Statistical Models During the Period of 1980 to 2010.” Science of the Total Environment 650, no. 1: 661–670. 10.1016/j.scitotenv.2018.08.398. 30212695

[ece371319-bib-0060] Wang, Y. , D. Peng , M. Shen , et al. 2020. “Contrasting Effects of Temperature and Precipitation on Vegetation Greenness Along Elevation Gradients of the Tibetan Plateau.” Remote Sensing 12, no. 17: 2751. 10.3390/rs12172751.

[ece371319-bib-0061] Wang, Z. , Y. Jia , P. Li , et al. 2023. “Study on Environmental Factors Affecting the Quality of Codonopsis Radix Based on MaxEnt Model and All‐In‐One Functional Factor.” Scientific Reports 13, no. 1: 20726. 10.1038/s41598-023-46546-6. 38007505 PMC10676394

[ece371319-bib-0062] Wen, J. , X. Lv , D. Hong , et al. 2016. “Potential Distribution of *Rhodiola crenulata* in Tibetan Plateau Based on Maxent Model.” China Journal of Chinese Materia Medica 41, no. 21: 3931–3936. 10.4268/cjcmm20162108. 28929677

[ece371319-bib-0063] Wu, X. , L. Zhong , G. Chen , et al. 2024. “Morphological and Physiological Plasticity of Alpine Medicinal Plants Along an Elevational Gradient.” Journal of Applied Research on Medicinal and Aromatic Plants 44: 100613. 10.1016/j.jarmap.2024.100613.

[ece371319-bib-0064] Xu, N. , F. Meng , G. Zhou , Y. Li , B. Wang , and H. Lu . 2020. “Assessing the Suitable Cultivation Areas for *Scutellaria baicalensis* in China Using the Maxent Model and Multiple Linear Regression.” Biochemical Systematics and Ecology 90: 104052. 10.1016/j.bse.2020.104052.

[ece371319-bib-0065] Xu, Z. , C. Zhao , and Z. Feng . 2009. “A Study of the Impact of Climate Change on the Potential Distribution of Qinghai Spruce (*Picea crassifolia*) in Qilian Mountains.” Acta Ecologica Sinica 29, no. 5: 278–285. 10.1016/j.chnaes.2009.09.004.

[ece371319-bib-0066] Yang, L. , X. Zhu , W. Song , X. Shi , and X. Huang . 2024. “Predicting the Potential Distribution of 12 Threatened Medicinal Plants on the Qinghai‐Tibet Plateau, With a Maximum Entropy Model.” Ecology and Evolution 14, no. 2: e11042. 10.1002/ece3.11042. 38362168 PMC10867876

[ece371319-bib-0067] Yang, X. , S. P. S. Kushwaha , S. Saran , J. Xu , and P. S. Roy . 2013. “Maxent Modeling for Predicting the Potential Distribution of Medicinal Plant, *Justicia adhatoda* L. in Lesser Himalayan Foothills.” Ecological Engineering 51: 83–87. 10.1016/j.ecoleng.2012.12.004.

[ece371319-bib-0068] Yao, Z. , Q. Han , and B. Lin . 2023. “Prediction of Distribution Area of Main Noxious and Miscellaneous Weeds in Xinjiang Based on MaxEnt Model.” Acta Ecologica Sinica 43, no. 12: 5096–5109. 10.5846/stxb202205061252.

[ece371319-bib-0069] Yi, Y. , X. Cheng , Z. Yang , Y.‐j. Yi , Z.‐F. Yang , and S.‐H. Zhang . 2016. “Maxent Modeling for Predicting the Potential Distribution of Endangered Medicinal Plant (*H. riparia* Lour) in Yunnan, China.” Ecological Engineering 92: 260–269. 10.1016/j.ecoleng.2016.04.010.

[ece371319-bib-0070] Yuan, X. , H. Wen , J. Zhao , et al. 2016. “Phytochemical and Chemotaxonomic Study on *Lagotis brevituba* (Scrophulariaceae).” Biochemical Systematics and Ecology 66: 8–11. 10.1016/j.bse.2016.02.025.

[ece371319-bib-0071] Zhan, P. , F. Wang , P. Xia , et al. 2022. “Assessment of Suitable Cultivation Region for *Panax notoginseng* Under Different Climatic Conditions Using MaxEnt Model and High‐Performance Liquid Chromatography in China.” Industrial Crops and Products 176: 114416. 10.1016/j.indcrop.2021.114416.

[ece371319-bib-0072] Zhang, D. , L. Tan , L. Yao , et al. 2020. “In Vitro and In Vivo Antioxidative Activity Against Radiation‐Induced Damage and the Systematic Chemical Components of Different Extracts of *Lagotis brevituba* Maxim.” Evidence‐Based Complementary and Alternative Medicine 2020, no. 1: 1–14. 10.1155/2020/9726431. PMC7758126 33381219

[ece371319-bib-0074] Zhang, Y. , S. Chen , Y. Gao , et al. 2023. “Prediction of Global Potential Suitable Habitats of *Nicotiana alata* Link et Otto Based on MaxEnt Model.” Scientific Reports 13, no. 1: 4851. 10.1038/s41598-023-29678-7. 36964182 PMC10038996

[ece371319-bib-0075] Zhang, Y. , J. Li , W. Lin , et al. 2011. “Prediction of Potential Distribution Area of *Erigeron philadelphicus* in China Based on MaxEnt Model.” Chinese Journal of Applied Ecology 22, no. 11: 2970–2976. 22303676

[ece371319-bib-0076] Zhao, J. , Z. Lancuo , W. Wang , et al. 2024. “Response of Suitable Area of Highland Barley in the Tibetan Plateau to Climate Change Based on Maximum Entropy Model Analysis.” Chinese Journal of Eco‐Agriculture 32: 1–13. 10.12357/cjea.20230665.

[ece371319-bib-0077] Zhao, M. , C. Peng , W. Xiang , et al. 2013. “Plant Phenological Modeling and Its Application in Global Climate Change Research: Overview and Future Challenges.” Environmental Reviews 21, no. 1: 1–14. 10.1139/er-2012-0036.

[ece371319-bib-0078] Zhao, W. , H. Chen , Y. Yuan , et al. 2021. “The Impact of Climate Change on the Distribution Pattern of the Suitable Growing Region for Tibetan Medicine *Lamiophlomis rotata* .” Acta Agrestia Sinica 29, no. 5: 956–964. 10.11733/j.issn.1007-0435.2021.05.012.

[ece371319-bib-0079] Zhao, Y. , X. Deng , W. Xiang , L. Chen , and S. Ouyang . 2021. “Predicting Potential Suitable Habitats of Chinese Fir Under Current and Future Climatic Scenarios Based on Maxent Model.” Ecological Informatics 64, no. 15: 101393. 10.1016/j.ecoinf.2021.101393.

[ece371319-bib-0080] Zhu, J. , Y. Shi , H. Wang , and M. Li . 2019. “Two New Phenylpropanoid Glycosides From *Lagotis Brachystachya* Maxim and Their Xanthione Oxidase Inhibitions.” Natural Product Research 35, no. 13: 2131–2136. 10.1080/14786419.2019.1662008. 31496304

[ece371319-bib-0081] Zhu, J. , H. Zhang , G. Zhong , and H. Wang . 2017. “Research Advances in Chemical Constituents and Pharmacology of Tibetan Herb *Lagotis* .” Chinese Journal of Experimental Traditional Medical Formulae 23, no. 12: 214–222. 10.13422/j.cnki.syfjx.2017120214.

